# Performance of machine-learning scoring functions in structure-based virtual screening

**DOI:** 10.1038/srep46710

**Published:** 2017-04-25

**Authors:** Maciej Wójcikowski, Pedro J. Ballester, Pawel Siedlecki

**Affiliations:** 1 Institute of Biochemistry and Biophysics PAS, Pawinskiego 5a, 02-106 Warsaw, Poland; 2 Centre de Recherche en Cancérologie de Marseille (CRCM), Inserm, U1068, Marseille, F-13009, France; 3 CNRS, UMR7258, Marseille, F-13009, France; 4 Institut Paoli-Calmettes, Marseille, F-13009, France; 5 Aix-Marseille University, UM 105, F-13284, Marseille, France; 6 Department of Systems Biology, Institute of Experimental Plant Biology and Biotechnology, University of Warsaw, Miecznikowa 1, 02-096 Warsaw, Poland

## Abstract

Classical scoring functions have reached a plateau in their performance in virtual screening and binding affinity prediction. Recently, machine-learning scoring functions trained on protein-ligand complexes have shown great promise in small tailored studies. They have also raised controversy, specifically concerning model overfitting and applicability to novel targets. Here we provide a new ready-to-use scoring function (RF-Score-VS) trained on 15 426 active and 893 897 inactive molecules docked to a set of 102 targets. We use the full DUD-E data sets along with three docking tools, five classical and three machine-learning scoring functions for model building and performance assessment. Our results show RF-Score-VS can substantially improve virtual screening performance: RF-Score-VS top 1% provides 55.6% hit rate, whereas that of Vina only 16.2% (for smaller percent the difference is even more encouraging: RF-Score-VS top 0.1% achieves 88.6% hit rate for 27.5% using Vina). In addition, RF-Score-VS provides much better prediction of measured binding affinity than Vina (Pearson correlation of 0.56 and −0.18, respectively). Lastly, we test RF-Score-VS on an independent test set from the DEKOIS benchmark and observed comparable results. We provide full data sets to facilitate further research in this area (http://github.com/oddt/rfscorevs) as well as ready-to-use RF-Score-VS (http://github.com/oddt/rfscorevs_binary).

Structure-based Virtual Screening (VS)^1^,^2^ aims at identifying compounds with previously unknown affinity for a target from its three-dimensional (3D) structure. Docking techniques are typically used to carry out this *in silico* prediction using their embedded scoring functions (SFs). When applied to VS, SFs seek to rank compounds based on their predicted affinity for the target as a way to discriminate between binders and non-binders. Despite the well-known limitations of SFs^1^,^3^,^4^,^5^, their application has been beneficial in many VS projects and successful applications have been reported^3^,^6^,^7^,^8^,^9^.

Although the classical SFs used in VS experiments have often proven useful, improved accuracy requires novel approaches. Usage of more than one SF to evaluate and rank ligands from chemical libraries is now standard practice in VS. Unfortunately, SFs do not account well for conformational entropy or solvation energy contributions, which is detrimental for binding affinity prediction^10^. Often, an empirical or knowledge-based SF is used to generate an ensemble of viable docking poses followed by a seemingly more rigorous energy-based SF, which is applied for re-scoring the poses to rank the corresponding ligands. The choice of appropriate SF is not obvious in such usage scenarios, since the predictive accuracy of a SF varies between protein families. SFs uniquely calibrated for the data set under study are often preferred to universal SFs^1^,^11^. Unfortunately, full training of classical SFs is often not possible. Many of them are provided in a way that does not permit changing the regression model, although a number of control parameters can be adjusted to tailor the SF to a particular target. Importantly, the underlying linear regression model employed by classical SFs has been shown to be unable to assimilate large amounts of structural and binding data^12^.

By contrast, machine-learning SFs provide clear advantages over these classical SFs^13^. Given a set of active and inactive ligands for training, SFs such as RF-Score^14^, NNScore^15^ and SFCscore^16^,^17^ can be trained to distinguish between known ligands by potency with high accuracy. Indeed, the degree with which machine-learning SFs have outperformed classical SFs at binding affinity prediction has been highlighted by several reviews^13^,^18^,^19^,^20^. Research has been carried out on various aspects of machine-learning SFs for binding affinity prediction: how target diversity affects predictive performance^21^, the impact of structure-based feature selection on predictive performance^22^, how to build machine-learning versions of classical SFs^23^, how predictive performance increases with the size of the training data in both types of SFs^12^, how the quality of structural and binding data influences predictive performance^24^, which machine learning (ML) methods generate more predictive SFs^25^, how to correct the impact of docking pose generation error on predictive performance^26^ or the implementation of webservers^27^ and stand-alone software^26^,^28^ to make these tools freely available. It is important to note that the validation of machine-learning SFs has generally been much more rigorous than that of most classical SFs^13^. For example, in building RF-Score v3 for binding affinity prediction, no overlapping between training and test sets is permitted by construction^12^. Importantly, any adjustable parameter of the machine-learning SF is selected from data not used to estimate the performance of the model^13^ (e.g. k-fold cross-validation^29^ is done for either model selection or estimating generalization performance, but not both). Typically, neither of these safeguards against model overfitting is enforced when measuring the performance of classical SFs^30^.

Machine-learning SFs have also shown advantages over classical SFs in structure-based VS. On retrospective VS studies, SVM-SP has strongly outperformed Glide, ChemScore, GoldScore and X-Score on HIV protease^31^. Another prime example is that of MIEC-SVM retrospectively outperforming Glide and X-Score^32^ on 40 DUD2 targets, in a study that also showed that VS performance increases with training set size as expected. This machine-learning SF has also been found superior to classical SFs in prospective VS studies on kinases^33^. It is still not clear however how different modelling choices affect predictive performance on particular targets. For instance, a recent study has found that the VS performance on HSP90 of a Neural Network-based SF is just comparable to that of Vina^34^.

In this work, we investigate what is the influence of including negative data instances (inactive molecules docked to targets) on machine learning SF. Such chimeric complexes are currently discarded from training procedures. Here we present an in-depth analysis of machine-learning SFs with respect to their classical counterparts, both in terms of VS and binding affinity prediction. We use the full DUD-E^5^ data sets for model building and performance assessment across 102 targets using three docking tools to generate the corresponding poses. Three machine-learning SFs using structural features with different degrees of complexity are used^12^,^14^,^22^ and compared to five classical SFs. We assess the VS performance of the SFs in both established-target and novel-target settings, either tailored for broad application or for a specific target.

## Materials and Methods

### Data provenance

Benchmarking platform Directory of Useful Decoys – Enhanced (DUD-E) resource was used to generate various classes of non-overlapping training and testing sets intended to simulate possible application scenarios (data accessed 01.08.2016). The data set consisted of 102 protein targets, with a group of active molecules for each target (224 ligands on average) and decoys (50 decoys per active ligand). A ligand is considered active if its affinity (IC_50_, EC_50_, K_i_, or K_d_) is 1 μM or better. DUD-E decoys comprise ligands selected based on similarity to physical properties of known actives (for a particular target), but dissimilar in terms of 2D-topology. Though the inactivity of decoys has not been experimentally confirmed, these are likely to be inactive and thus this procedure minimizes the risk of selecting false negatives^5^.

### Training and test sets

Classical SFs are used off-the-shelf on the DUD-E benchmark test sets, which means that there might be a number of protein-ligand complexes in common with their training sets. However, this overlap should be small as these training sets contain at most a few hundred complexes (this is because the underlying linear regression model cannot learn beyond this point^12^). By contrast, machine learning is able to exploit much larger training data sets, which would lead to a much larger overlap. Consequently, we use cross-validations on the DUD-E sets to avoid any protein-ligand complexes in common between training and test sets.

**Figure 1:**
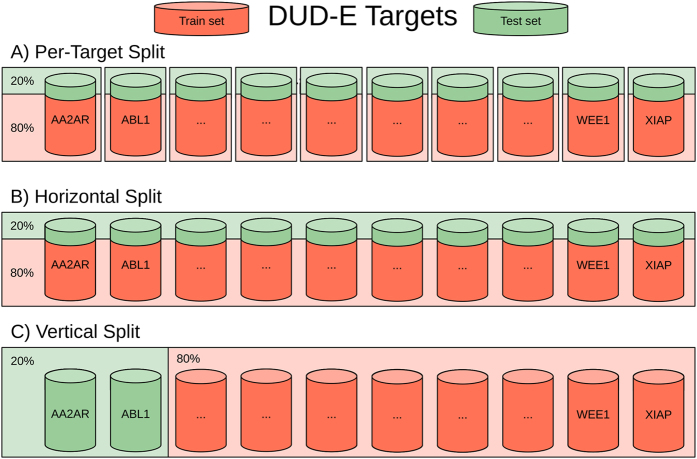
Per-Target, Horizontal and vertical split of DUD-E targets. Each barrel represents all the protein-ligand complexes (actives and decoys) associated with a different target. The training sets are coloured red, the test sets with green.

With the purpose of representing different common scenarios with respect to how much data is available for the target, we have introduced three different kinds of stratified 5-fold cross-validations (see Fig. 1) (1) **per-target –** in this approach, we generate 102 unique machine-learning SFs, each created independently for a single protein target (trained only on its active and decoys ligands); (2) **horizontal split –** both training and test sets contain data from all targets, i.e. each target has its ligands both in training and test sets. Such approach mimics experiments where docking is performed on targets for which there are already known ligands; (3) **vertical split –** the training and test data are created independently, i.e. there are no shared targets between training and test data. The vertical split represents the scenario, where SFs estimate whether a molecule binds to a target with no known ligands. As both machine-learning and classical SFs employ regression models, each SF returns a predicted real-valued score for each DUD-E protein-ligand pair that is thereafter used to rank them prior to apply the inactive cutoff and evaluate classification performance.

### Validation

The k-fold cross-validation (CV) is a commonly used strategy to reduce model overfitting. It also serves to assess applicability and generalization of predictions. Throughout this work we used 5-fold CV, which corresponds to an 80:20 test and training set ratio. This means that 80% of the data is used for training the machine-learning SF, which is afterwards tested on the remaining 20%. Such cross validation is repeated 5 times, meaning the whole dataset is divided into 5 groups (folds) of target-ligand complexes of approximately equal size. The folds are stratified – the proportions of actives/inactives preserved from original dataset. The function is learned on 4/5 of the groups and tested on 1/5 of the remaining complexes (on average). Importantly, each protein-ligand complex was present in training and test sets at some point, but never in both sets concurrently (i.e. in a single fold). This way training and test sets never overlap.

### Data normalization and compounds labelling

Molecules from DUD-E are considered inactive when their activity on the target is weaker (i.e. lower) than pK_d/i_ = 6 (this is the same activity cutoff which was used by the DUD-E authors^5^). In order to adapt RF-Score to VS, its regression model needs to be trained on a large proportion of inactive protein-ligand pairs. Therefore, all training decoys were assigned an identical inactive value of pK_d/i_ = 5.95 (less than 1% under the activity cutoff). We have also tested lower inactive cutoffs, also reported elsewhere^18^, but found these to be suboptimal (data not shown). Following common convention, data points were converted to pK_d/i_ units (this customary step had no significant impact on predictions made by the SFs).

### Ligand poses generation

Ligand 3D conformations were generated with three docking programs; AutoDock Vina (the Smina implementation, [http://smina.sf.net/])^35^,^36^, Dock 3.6^37^ and Dock 6.6^37^,^38^,^39^. Dock 3.6 was not run in-house, instead the DUD-E docked conformations and scores were downloaded from the database website (http://dude.docking.org). Vina and Dock 6.6 software were used with default parameters. Target proteins were prepared using UCSF Chimera^23^ DockPrep tool. Docking box was constrained to 10 Å around the ligand (which was included in the crystallographic structure of every protein target). Ligands were prepared and sanitized using OpenBabel^40^. 50 conformations were generated with each docking methodology.

### Classical Scoring Functions

Ligand-receptor complexes were scored using several classical, commonly used SFs. We used internal SFs implemented in the three docking programs used (Vina, Dock3.6, Dock6.6). Additionally we used the CScore module of SybylX 2.1^41^ which implements: D_Score^38^, G_score (known as GoldScore^42^), ChemScore^43^ and PMF_Score^44^. All of the above SFs have been widely used in VS.

### Descriptors and machine learning models

RF-Score is currently one of the best performing SFs at binding affinity prediction^12^,^13^. We used the descriptors from RF-Score versions v1^14^, v2^22^ and v3^12^. All versions use the same distance cutoff; a pair is tallied as interacting when the distance between the atoms falls within the 12 Å cutoff. They differ in the number of bins used. Bins divide the basal cutoff into smaller ranges, e.g. 2 Å bins (used by v2) translates to bins of following sizes: 0–2 Å, 2–4 Å, 4–6 Å, 6–8 Å, 8–10 Å, 10–12 Å. Versions v1 and v3 use only one bin. Finally, v3 is enriched with Autodock Vina partial scores.

Each RF-Score-VS version was trained on one best scoring ligand pose (meaning the lowest score from corresponding docking software). The number of trees in random forest (RF) was set to 500 as in the original implementation (this setting has been shown to be robust^45^). The number of features to consider when looking for the best split in each RF tree (“mtry”) was optimized using out-of-bag predictions (OOB). The optimized values are: 15 for v1 and v3 and 100 for v2 used in this study (i.e. we do not tune RF to DUD-E data). All RF-Score-VS calculations were done using ODDT^28^.

### External dataset validation

DEKOIS 2.0^3^ database was used as external validation dataset. Four overlapping structures between DUD-E and DEKOIS 2.0 were filtered out: A2A: “2p54”, HDAC2: “3l3m”, PARP-1: “3eml”, PPARA: “3max”. Protein SIRT2 had no crystal ligand, thus was also excluded. The final, non-overlapping dataset consisted of 76 targets. In addition, we have filtered out any ligand or decoy, which was found to be nearly identical (Tanimoto score of at least 0.99; OpenBabel FP2 fingerprints) to any ligand/decoy present in DUD-E. Originally each DEKOIS protein was associated with 40 ligands and 1200 decoys, our pruning removed on average 18.6 (46.5%) ligands and 188 (15.7%) decoys.

Protein files were prepared according to DEKOIS 2.0 publication^3^ using Schrödinger Maestro suite. Afterwards ligands and decoys were docked using Autodock Vina with default settings, as previously done with DUD-E. Docked poses were rescored with RF-Score-VS v2 and v3 scoring functions.

### Metrics

Enrichment factor (EF) is a fraction of active molecules within a given percentile of ranking list divided by random hit-rate. In DUD-E database there are 50 decoys per active ligand, hence random hit-rate is ~2%. DEKOIS database after pruning on average contains 21 actives and 1012, which translates to the same ~2% random hit rate as in DUD-E. Enrichment Factor, the area under Receiver Operating Characteristic curve (ROC AUC) and other metrics were calculated using ODDT^28^.

## Results and Discussion

The aim of the work was to mimic VS campaigns using diverse targets having a number of active and inactive ligands. It is well known that in practice the number of inactives of a target is much larger than that of actives. DUD-E dataset^5^ was built primarily to test performance of docking and scoring software, but it also fits into the constrains of a screening dataset described above. It contains of 102 targets associated with 22,886 ligands with measured activity. The target types are quite diverse and consist of receptors (GPCR, chemokine and nuclear), globular enzymes, kinases and virus proteases among others. It is also heterogeneous in case of ligand abundance; Catechol O-methyltransferase (COMT) has only 41 active compounds, compared to MAP kinase p38 alpha (MK14) which has 578 unique, dissimilar compounds. On average there are 224 ligands per target and for each of them 50 decoys were generated according to procedure described by Mysinger *et al*.^5^. These decoy compounds are presumed inactive because their chemical structures are dissimilar to those of known ligands. However, they are designed to share the same physiochemical features (number of donors/acceptors, etc.), so that discrimination between actives and inactives is not trivial. So in this setup, an average screening campaign would search through 11 200 compounds to find 224 active ligands.

The most common measure of retrospective VS performance is the enrichment factor (EF) of a method applied to a particular benchmark. When a large database of compounds is screened one takes the best scored compounds at the top of the ranked list for further evaluation. The number of experimentally tested compounds is chosen depending on various criteria, but it is usually the top of the list which is pursued (e.g. top 1%, 0.1%, etc.). Therefore, it is not the overall performance of a scoring method on the whole database, such as ROC AUC, which is most relevant for VS, but rather the performance in the top of the list, i.e. how many active compounds are among the best scored compounds. In our assessment, we focused on EF_1%_ (fold change of active molecules percentage within the top 1% of ranking list over random distribution) as the most relevant estimate of screening performance and machine learning predictive power.

We perform a stratified 5-fold cross validation (see materials and methods for more details), to avoid model overfitting which hampers the performance on data sets other than the training set^46^. It is important to note that, while all target-ligand complexes are present at some point in training and test sets, they are never in both simultaneously (see Materials and Methods, validation section). Finally, the mean value of the performance in 5 independent folds is calculated, which estimates how a model will perform on independent datasets. This prevents testing the SF on complexes used for training and reporting artificially boosted performance.

The first experiment we conducted was to train the SFs on **horizontally split data**. This approach mimics experiments where docking is performed on targets for which there are already known active ligands and VS is done to find new ones. Therefore, training was done on 4/5 of ligands from all 102 DUD-E targets and the model was tested on the remaining 1/5 (Fig. 1B). In this setup only a single, unique model (generic SF) is built for the whole DUD-E dataset. Such model can be directly compared to a classical SF as they are also single model functions, trained on a defined set of protein-ligand complexes and developed to work with diverse targets.

**Figure 2:**
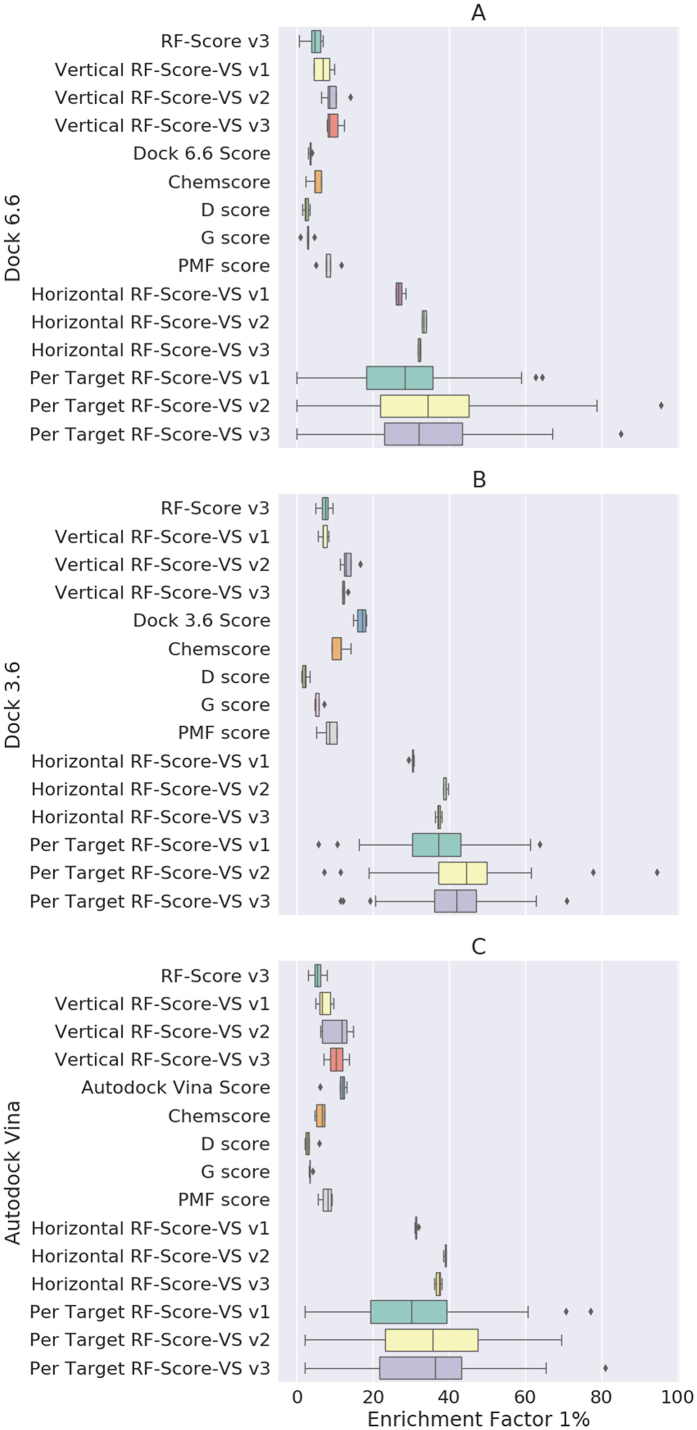
Comparison of EF_1%_ results obtained from classical SFs: D_score, Chemscore, G_score, PMF_score, native score (i.e. which was used to by docking software), with results from three versions of RF-Score-VS. Unlike RF-Score-VS, RF-Score v3 does not train on any negative data (this SF for binding affinity prediction was exclusively trained on X-ray crystal structures^12^). Each boxplot shows five EF_1%_ values for a given SF resulting from the five 80:20 data partitions (i.e. five non-overlapping test sets collectively comprising all data). All train-test splitting scenarios are present, namely vertical, horizontal and per-target. A dramatic increase in machine-learning scoring performance (measured as EF_1%_) can be seen in RF-Score-VS compared to classical SFs.

Our results show a dramatic increase of EF_1%_ performance between the best classical compared to machine-learning SF trained on horizontally split dataset: around two- to even 15-times increases depending on the docking engine and SF (Fig. 2). It is worth noting that the classical SF do not perform similarly here, as the obtained EF_1%_ varied significantly both in value and standard deviation. RF-Score v3, which is one of the best performing machine-learning SF to predict binding affinities on PDBbind, yields EF_1%_ similar to best performing classical approaches despite being trained on X-ray crystal structures and thus not incorporating any negative data (i.e. docked inactives) into the training set. In contrast, novel machine-learning methodology was much more robust in terms of protein-ligand complexes provided by the three docking algorithms. Independently from the docking engine the EF_1%_ values for the developed SFs osculated mostly well above 30. The worst machine-learning screening combination (Dock 6.6 and RF-Score-VS v1) was still almost two-times better than the best performing classical combination (Dock 3.6 and its native SF). This trend holds even for smaller top percentages. EF_0.1%_ for the best performing classical Dock 3.6 compared to horizontal RF-Score-VS v2 is twofold smaller (29.39 to 61.42). See Supplementary File for more results comparison.

As expected, when looking more deeply into the obtained data it was clear that results from different targets can vary significantly. There are targets that seem to be hard for the SF; defined as those with EF_1%_ < 20. On the other end, there are also easy targets, the SF showing outstanding performance, i.e. EF_1%_ > 60. There was not obvious correlation between the number of active ligands among hard or easy targets. What we found however is that the hard targets are difficult very much independently from the employed docking software or ML training approach. Thus, the problem might be due to to inaccurate 3D representation of receptor-ligand complexes or inappropriate choice of binding site for some ligands.

**Figure 3:**
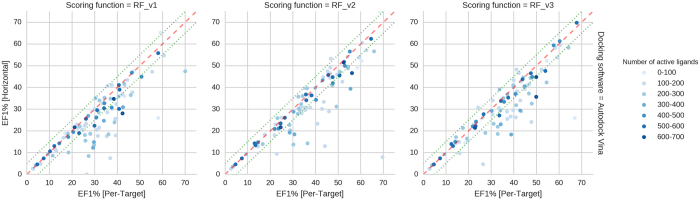
Comparison of EF_1%_ results from Per-Target and Horizontal-split models. Each data point is a separate corresponds to the performance of both models on a particular DUD-E target. The darker is the colour of DUD-E target is, the more active ligands it has. Docking conformations were obtained from Autodock Vina. Dashed red line denotes equal performance, and dotted green line show 5-unit intervals. For most targets and contrary to common assumption, there is little advantage in training machine machine-learning SFs for per-target vs using a more generic approach (in this case horizontal split), especially for targets with greater number of active molecules.

We also investigated the question: whether it is beneficial to train machine-learning SFs only on data specific to a particular target and then use it for screening rather than using a generic function trained on all targets. Put differently: do tailored functions perform significantly better than a generic function to justify the additional effort undertaken for training? To answer these questions, we trained a separate SF for each of the DUD-E targets and compared its performance with the generic SF (obtained with horizontal split sketched in Fig. 1). The results of these experiments are presented in Figs 2 and 3. On Fig. 3, we demonstrate pairwise comparison of per-target and horizontal SFs. Every point is a cross-validated predictions for single DUD-E protein.

To our surprise most of the per-target functions tend to perform only slightly better than the generic, unique function, trained on all available data. Almost 2/3 of targets (64 out of 95 – some targets failed to dock with default Vina settings, thus 95 not 102) had its EF_1%_ increased less than 10%, regardless of the docking program used. As before, this is tested with 5-fold cross-validation and an average of test splits is the final result. Contrary to common assumption there was little advantage in training machine learning scoring for most of the targets vs using a single generic approach (trained on the horizontal split dataset). This was especially visible for targets with greater number of active molecules.

In the case of hard targets, most of them did not improve by per target training. However, a subset of hard targets, generally having a lower number of active molecules (between 1 and 200), seem to benefit from such per-target training. Figure 3 shows clearly that in those cases per target training can improve performance significantly. This result might come as a surprise, as in principle if a target has less data to train on, then it should be better predicted using additional data from other targets’ complexes. Figure 3 shows this is usually not the case. One explanation of these results could be that per-target training is done on a small but very specific set of interactions; These might be much more important for this particular target, but their low abundance in others can decrease their weight in a generic (horizontal) function. Can we improve the performance of hard targets by using additional data, but only from targets with similar active site structures? Such questions are still open.

Finally, we look at the question of how suitable machine-learning scoring is for newly discovered targets characterized by scarce data on active ligands. To answer this question, the training and test data are created independently, i.e. there are no shared targets between training and test data. We call this experiment the “Vertical split”, where machine learning SFs were not trained with any complex involving the target of interest.

As expected this significantly influences the results. Figure 2 shows that there is a drop in EF_1%_ performance between horizontal SF (which was oscillating around 35–40 and more) and Vertical RF (which is in the area between 10 and 15). The results from different versions of RF-Score descriptors were also less robust to the influence of conformations provided by docking engines. Nevertheless, this dispersion is still smaller than obtained from a classical approach.

In the case of Dock 6.6 (Fig. 2A), the best classical SF (PMF-Score) obtained an EF_1%_ comparable to vertical RF-Score-VS v1. RF-Score-VS v3 however performed significantly better than PMF, twofold better compared to the second best ChemScore function (11.4 vs. 4.9 respectively) and 3 to 4-times better than the remaining three functions (D-Score, G_Score and Dock 6.6 built-in function). In fact, a similar pattern can be observed with all three docking algorithms (Fig. 2B and C); ChemScore and PMF-Score are the best performing classical SFs with EF_1%_ comparable to vertical RF-Score-VS v1. Nevertheless, RF-Score-VS v2 and v3 outperform all classical approaches in this scenario. Interestingly, the EF_1%_ value obtained with Dock3.6 and its built-in native function (Fig. 2B, Dock 3.6 Score) was unexpectedly high in comparison with other classical SFs. Dock3.6 was the only SF that we did not run ourselves, as both its docked molecules and predicted scores were downloaded directly from DUD-E website. Therefore, we assume that there was some kind of tailored procedure for each target prior or after docking, which is a potential source of overfitting. In our procedure (as described in the materials and methods section), we have not done any work on the receptor nor ligand datasets. In addition, we have only used the default settings for all the SFs that we have tested, including the two other docking algorithms Dock 6.6 and Autodock Vina.

The results obtained from the vertical split experiments show that machine-learning SF (such as RF-Score-VS v3) trained on data from other targets is: 1) able to outperform the five tested classical SF without the need for any calibration steps and 2) less sensitive to docking conformation than the classical SFs. These results are relevant for the case where *in silico screening* is used with a novel target with no known active ligands. As shown, using RF-Score-VS v3 would make screening much more simple (no need to use different docking, tailor them to each target or SF) and still produce better results.

In the case of Dock 3.6 results, it would seem that a manually calibrated VS experiment using carefully chosen classical SF may be capable of performing as well as RF-Score-VS v3 when absolutely no complex of the predicted target is used. This is a rare situation that simulates structure-based VS on completely new targets. For such targets there is simply not enough data to properly calibrate the classical procedure, and so there is no simple way to make an educated guess on which combination of docking parameters and SFs to choose. In fact, the Dock 3.6 case is an example where model selection and performance measurement are both carried out on the training data, which is known to lead to an unrealistically high estimation of the generalization error of the model (in this context, how well the SF with exactly the same settings will rank other molecules docked to the same target). It is therefore worth mentioning that even in such extreme setup as described above (the control parameters tuned on data not available to machine-learning procedure) RF-Score-VS v3 results were still comparable to the overfitted classical SF.

### Validation on an independent test set

We have also tested RF-Score-VS trained with the entire DUD-E (15 426 active molecules and 893 897 inactive molecules across 102 targets) on an external data set. Such data set is hard to find as either most of the targets overlap between sets or they provide a small number of ligands per target. In contrast, the DEKOIS 2.0 benchmark overcomes both obstacles: only 4 of the 81 DEKOIS targets are also in the DUD-E benchmark (see Methods section for further details).

**Figure 4:**
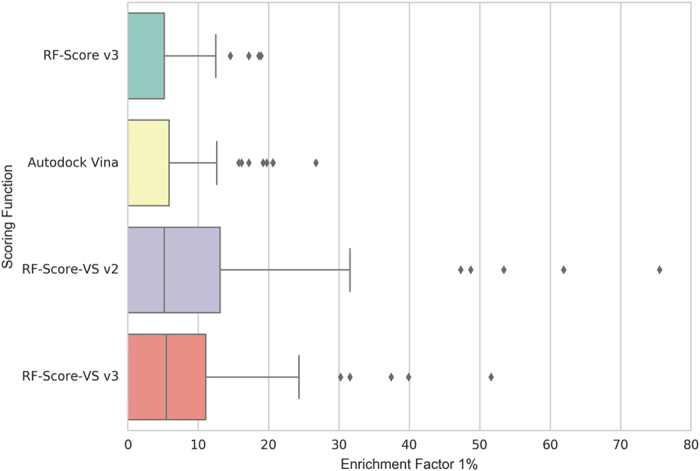
Boxplots presenting EF_1%_ for Autodock Vina, RF-Score v2 and novel RF-Score-VS v2 and v3 training on negative data on the part of the DEKOIS 2.0 benchmark not overlapping with DUD-E benchmark (i.e. different targets, ligands and decoys).

The results of early enrichment for 76 DEKOIS targets are summarized on Fig. 4. Autodock Vina, which in previous experiments was the best scoring function run in-house, achieved EF_1%_ = 3.95 and RF-Score v3 scored EF_1%_ = 2.94. These results are in line with what was obtained with DUD-E data. By contrast, RF-Score-VS v2 and v3 performances were EF_1%_ = 9.84 and EF_1%_ = 7.81, respectively, thus more than doubling the active compounds yield in the top 1%. Despite the achieved improvement, structure-based VS remains a challenging problem on a number of targets, where all three SFs fail to find any active within the top 1% of their ranked lists. Therefore, it is clear that more work is needed to advance further in this problem.

### Top results analysis

**Figure 5:**
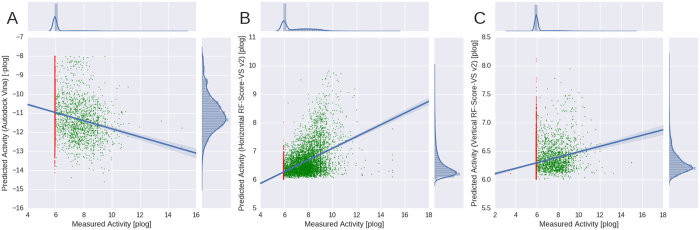
Predicted vs measured activity. Top 1% of compounds predicted to be active for each target in DUD-E by (**A**) the Autodock Vina and its native SF (R_p_ = −0.18); (**B**) RF-Score-VS v2 trained on horizontally split dataset (R_p_ = 0.56); and (**C**) RF-Score-VS v2 trained on vertically split dataset (Rp = 0.2). Red points represent decoys (putative inactive compounds), green points – compounds with measured activity. Predicted values for machine-learning SFs are taken from the relevant cross-validation split.

Boxplots in Fig. 2 show summaries of the classification data. If a compound was in the top 1% of ranking list and it was active the enrichment factor (EF_1%_) value increased, if it was a decoy (presumed inactive) EF_1%_ decreased. These plots however do not show if these top 1% molecules are actually the most active ones. Thus, we can check whether machine-learning methodology predict binding affinity better than a classical SF. The scatter plots presented in Fig. 5 address these questions. We took the original 1% of the best *in silico* predictions for each DUD-E target (i.e. on average about 86 compounds per target) and assessed how their scores correlate with experimentally measured activity (derived from DUD-E database per-target).

Figure 5A displays the results obtained with the best classical SF, among those ran in-house (Autodock Vina and its native SF) compared to two training versions of RF-Score-VS: the generic (horizontal split) function Fig. 5B and to the novel target (vertical split) function Fig. 5C. The results indicate a clear advantage of score-affinity correlation for both training cases, compared to the best classical approach. Autodock Vina and its native SF obtains a Pearson correlation of R_p_ = −0.18 (this is actively a positive correlation as low Vina scores aim at being indicative of high binding affinity), where RF-Score-VS v2 horizontal split receives an impressive R_p_ = 0.56. Even in the less favourable scenario, the obtained vertical split is already R_p_ = 0.2. This can also be seen on the supporting ordinate and abscissae plots where compounds distribution resembles a normal distribution, while with classical SF the decoys clearly overwhelm the actives counts.

More importantly, machine-learning SFs trained with a high proportion of inactive instances (red dots) are much better at discriminating between actives (green dots) and inactives (red dots). For example, in Fig. 5A many inactives are nevertheless predicted to be active by Vina, but this is not the case with horizontally-trained RF-Score-VS v2. In the Vertical split more decoys are present but still much fewer than with classical approach and only few of them have high predicted affinities. When taken together, in the top 1% of all target screens, the horizontal split RF-Score-VS v2 obtained 55.6% (4875/8816) active compounds, whereas in the classical approach only 16.2% (1432/8816) of compounds where active. Moreover, the proportion of actives of RF-Score-VS is even more impressive with 88.6% (825/931) for the top 0.1% results, a much higher hit rate than that of Vina (27.5%; 256/931).

These results demonstrate how large is the improvement introduced compared to a widely-used classical SF– the novel approach has over three times greater yield of active ligands.

## Conclusions

The presented analysis demonstrates that previously implemented machine-learning SFs using RF-Score descriptors can excel at VS, if appropriate care is taken. Several cross-validation scenarios show that in any application RF-Score-VS comfortably outperforms classical SFs, even when using the most crude RF-Score v1 features^14^,^22^.

We report average enrichment factors (EF_1%_) across DUD-E targets to be 39 for generalized- (horizontal split) and 43.43 for specialized SF (per-target model), whereas the best classical approach (Dock 3.6) yields EF_1%_ = 16.86. This result translate to over 2.2 fold improvement in early enrichment showing exceptional advantage of RF-Score-VS in VS. Discriminating between actives and inactives is not the only task at which our proposed methods excel, as we also show the scoring and ranking power of our novel method. Pearson correlation of RF-Score-VS is three times better at reproducing top scored affinities (R_p_ = 0.56 for RF-Score-VS v2 vs R_p_ = −0.18 for Autodock Vina). For smaller top percentage, i.e. 0.1%, hit-rate advance of RF-Score-VS is even more evident −88.6% vs 27.5%.

In addition, we present results evidencing that it is not true that SFs based on RF-Score descriptors are “unable to enrich virtual screening hit lists in true actives upon docking experiments”^46^. We also comment on a statement made in a recent review^47^, where a 10% hit-rate was considered to be an upper limit to what SFs can nowadays deliver. In this study, RF-Score-VS achieves a hit rates as high as 88.6% across DUD-E targets, which is an outstanding performance. In addition to VS performance, we also show that docking equipped with cutting-edge machine-learning SFs will predict binding affinity accurately (R_p_ = 0.56) in the context of structure-based VS.

Developing new descriptors and validating other models is out of scope of this publication. However, we acknowledge that RF-Score v1 descriptors are not optimal, e.g. the generous 12 A cutoff might be in fact less sensitive to detecting subtle structural changes in protein-ligand complex. RF-Score v1 descriptors were only intended to show that the sophisticated descriptors that have dominated the research in this area generally add very little to performance, as it can be clearly seen here in terms of VS and binding affinity prediction. On the other hand, supplementing v1 descriptors with Vina partial scores is in most cases as beneficial as using v2, therefore a combination of them might be the most fruitful.

Research on the optimal application of machine learning to structure‐based VS is highly promising, but it is still in its infancy due to being a more complex endeavour than binding affinity prediction from crystal structure of protein-ligand complexes. Indeed, training data sets for structure-based VS are much larger than those used binding affinity prediction and require prior docking of each considered molecule. Future work in this area is expected to yield particular insight in terms of improving our ability to discriminate between actives and inactives across targets. Even larger amounts of data can be used following the described procedure. Many other machine learning techniques can be applied to structure-based VS. This is not only restricted to regression techniques, but also classifiers. For example, state-of-the-art multi-category classifiers^48^. Another promising avenue for future research is feature selection, not only in terms of the performance improvement but also considering the stability of the predictors^49^.

All data (docked poses) and workflow scripts required to recreate the generation of descriptors and training of machine-learning models are released here to the relevant research communities, making our software reproducible and for others to build upon it (http://github.com/oddt/rfscorevs). We also propose a standalone machine-learning based SFs RF-Score-VS v2 and v3, as a general purpose and target independent VS tools. To the best to our knowledge, RF-Score-VS is the best performing SF in terms of early enrichment EF_1%_ on DUD-E. RF-Score-VS can be downloaded from https://github.com/oddt/rfscorevs_binary (it is provided as standalone binary for Windows, Mac and Linux without any further dependency, with wide range of supported molecular formats). Alternatively RF-Score-VS may be used with ODDT toolkit environment as a drop-in replacement for any other SF in custom workflows and other software.

## Additional Information

**How to cite this article**: Wójcikowski, M. *et al*. Performance of machine-learning scoring functions in structure-based virtual screening. *Sci. Rep.* **7**, 46710; doi: 10.1038/srep46710 (2017).

**Publisher's note:** Springer Nature remains neutral with regard to jurisdictional claims in published maps and institutional affiliations.

## Supplementary Material

Supplementary Table 1
